# Novel Compound Heterozygous Mutations in *CRTAP* Cause Rare Autosomal Recessive Osteogenesis Imperfecta

**DOI:** 10.3389/fgene.2020.00897

**Published:** 2020-08-14

**Authors:** Yen-An Tang, Lin-Yen Wang, Chiao-May Chang, I-Wen Lee, Wen-Hui Tsai, H. Sunny Sun

**Affiliations:** ^1^Center for Genomic Medicine, Research and Service Headquarter, National Cheng Kung University, Tainan, Taiwan; ^2^Institute of Molecular Medicine, College of Medicine, National Cheng Kung University, Tainan, Taiwan; ^3^Division of Hematology Oncology, Department of Pediatrics, Chi Mei Medical Center, Tainan, Taiwan; ^4^Department of Childhood Education and Nursery, Chia Nan University of Pharmacy and Science, Tainan, Taiwan; ^5^School of Medicine, College of Medicine, Kaohsiung Medical University, Kaohsiung, Taiwan; ^6^FMC Fetal Medicine Center, Tainan, Taiwan; ^7^Division of Genetics and Metabolism, Department of Pediatrics, Chi Mei Medical Center, Tainan, Taiwan; ^8^Graduate Institute of Medical Sciences, College of Health Sciences, Chang Jung Christian University, Tainan, Taiwan

**Keywords:** whole-exome sequencing, *CRTAP*, skeletal dysplasia, osteogenesis imperfecta, splicing-altering variant

## Abstract

Whole-exome sequencing (WES) has advantages over the traditional molecular test by screening 20,000 genes simultaneously and has become an invaluable tool for genetic diagnosis in clinical practice. Here, we reported a family with a child and a fetus presenting undiagnosed skeletal dysplasia phenotypes, while the parents were asymptomatic. WES was applied to the parents and affected fetus to identify the genetic cause of the phenotypes. We identified novel compound heterozygous mutations consisting of a single-nucleotide variant (SNV) and a large deletion in the *CRTAP* gene (NM_006371.4:c.1153-3C > G/hg19 chr3:g.32398837_34210906del). Genetic alterations of *CRTAP* are known to cause osteogenesis imperfecta (OI) in an autosomal recessive manner. Further examination of the proband’s elder sibling who was diagnosed as OI after birth found that she shares the inherited compound heterozygous mutations of *CRTAP*; thus, the findings support the disease-causing role of *CRTAP* mutations. Through the *in vitro* molecular test and *in silico* analysis, the deleterious effects of the splicing-altering SNV in *CRTAP* (c.1153-3C > G) on gene product were confirmed. Collectively, our WES-based pathogenic variant discovery pipeline identifies the SNVs and copy number variation to delineate the genetic cause on the proband affected with OI. The data not only extend the knowledge of mutation spectrum in patients with skeletal dysplasia but also demonstrate that WES holds great promise for genetic screening of rare diseases in clinical settings.

## Introduction

Skeletal dysplasias are a series of heterogeneous genetic disorders that affect primarily cartilage and bone but also have a significant effect on joints and muscles (Krakow, 2015). According to the 2019 Nosology and Classification of Genetic Skeletal Disorder, skeletal disorders comprise 461 different conditions that are sorted into 42 groups based on their clinical and/or molecular phenotypes (Mortier et al., 2019). Among them, 425 conditions are shown to be associated with pathogenic variants in one or more of 437 different genes. Mutations in different loci within one gene can cause distinct types of skeletal disorders, while mutations in different genes can lead to the same disease (Foldynova-Trantirkova et al., 2012; Maioli et al., 2019). Therefore, genetic testing is indispensable in conjunction with clinical examination and radiographs for diagnostic confirmation. The encoding proteins of those disease-causing genes have diverse functions involving different signaling pathways, such as NOTCH, BMP, or TGFβ signaling, which are essential for skeletal development, growth, or homeostasis (Krakow, 2015). Interestingly, some pathogenic variants in mitochondrial-associated proteins have been shown to cause skeletal dysplasias, which is not common for mitochondria-related disorders (Mehawej et al., 2014; Dikoglu et al., 2015; Girisha et al., 2019; Peter et al., 2019). In addition to the protein-coding gene, the epigenetic alteration, first included in 2019 Nosology, indicates a novel gain-of-function variant in microRNA miR-140, which causes autosomal dominant skeletal dysplasia (Grigelioniene et al., 2019; Mortier et al., 2019). Although there has been substantial progress in understanding the genetic landscape of skeletal dysplasias, many more genes or mutations may be involved given the complicated phenotypes and the heterogeneous nature of the disorders. Given this complexity, genetic diagnosis of skeletal dysplasia has been a great challenge due to the involvement of a variety of genetic alterations (Zabel and Spranger, 2017; Mortier et al., 2019).

Among all skeletal disorders, osteogenesis imperfecta (OI) is the most common one that presents with decreased bone density, and the severe forms may present in the newborn period with fractures sustained *in utero* (Krakow, 2015). OI is a collective term for a group of rare connective tissue disorders that share similar skeletal deformities and are characterized by low bone mass and extremely fragile bones. Due to its phenotypical and genetic heterogeneity, recent nosology has classified OI into types ranging up to XIX (Mortier et al., 2019; Rossi et al., 2019). The incidence of OI varies across different populations, ranging from 0.3 to 1.5 per 10,000 births (Orioli et al., 1986; Kuurila et al., 2002; Stevenson et al., 2012; Folkestad et al., 2016), with the majority of defects in type I collagen structure and synthesis. Autosomal dominant mutations in *COL1A1* and *COL1A2*, coding for the α1(I) and α2(I) chains of type I collagen, account for 85–90% of all cases (Van Dijk and Sillence, 2014). In addition to the autosomal dominant inheritance, mutations in the genes involved in collagen-related metabolic pathways, such as posttranslational modification, folding and cross-linking, bone mineralization, and osteoblast differentiation with collagen insufficiency, are known to cause an autosomal recessive form of OI (Barnes et al., 2006; Forlino and Marini, 2016). Cartilage-associated protein (CRTAP), which forms a trimeric complex with P3H1 and cyclophilin B for 3-hydroxylation of proline 986 residue of collagen α1(I) and proline 707 residue of collagen α2(I) (Morello et al., 2006; Mehawej et al., 2014), respectively, was found to cause an autosomal recessive form of OI (Morello et al., 2006; Girisha et al., 2019). *CRTAP* deficiency leads to disruption of chain alignment and helical folding and results in defects of collagen synthesis (Pyott et al., 2011). The affected OI patients who were attributed to *CRTAP* mutations have been classified as type VII in the Online Mendelian Inheritance in Man (OMIM) database (OMIM #610682). Notably, the symptom and severity of OI are highly heterogeneous from case to case, even among individual patients carrying the same variant within the family (Forlino and Marini, 2016). Therefore, among the clinically indistinguishable cases, especially in the perinatal period, the genetic testing of OI is a critical and necessary step for diagnostic confirmation (Marini et al., 2017).

The traditional genetic carrier screening is based on an individual’s ethnic background or family history and is offered with a limited number of causative mutations leading to the specific diseases (Lazarin et al., 2013). This strategy has some restrictions, especially in the situation where the proband’s phenotype is not well defined and/or the causative genetic defects to the disease are highly heterogeneous. Therefore, expanded carrier screening has been recommended and requires a more comprehensive technique to identify carriers for a broader array of diseases (Edwards et al., 2015; Antonarakis, 2019). With the advances of sequencing technology over the past decade, whole-exome sequencing (WES) shows the potential to be widely used in the procedure of expanded carrier screening in a clinical setting, especially for rare diseases that have undergone exhaustive evaluations and for which an etiologic diagnosis is still unable to be provided (Posey et al., 2016; Sawyer et al., 2016). WES screens thousands of genes simultaneously by sequencing DNA in the protein-coding regions of an individual’s genome (also known as exomes). Although WES sequence accounts for less than 2% of the entire human genome, it contains more than 85% of known disease-causing variants (Sims et al., 2014; Salk et al., 2018). Importantly, in addition to single-gene Mendelian disorders, several studies recently showed that WES enables identification of multilocus pathogenic variations in families with complex clinical indications and/or variable severities (Karaca et al., 2018; Alves et al., 2019). Results from these studies further highlight the promising land of WES in genomic medicine.

Here, we report an affected fetus with complex and undiagnosed skeletal phenotypes, short long bones and multiple severe fractures, while the parents are phenotypically asymptomatic. We applied the WES pipeline and identified that the proband harbored a novel compound heterozygous single-nucleotide variant (SNV) and deletion in the *CRTAP* gene. The mutations were validated by Sanger sequencing (Sanger-seq) and array comparative genomic hybridization (aCGH). The proband’s elder sibling, who was diagnosed as OI, was also confirmed to have the compound heterozygous mutations in the *CRTAP* gene. In particular, we uncovered the novel splicing variant in the *CRTAP* gene as a deleterious mutation by showing the reduced mRNA level as a consequence of mutation-mediated mRNA decay. We further assessed and confirmed the pathogenicity of the novel compound mutations in *CRTAP* that cause OI. Our findings uncover novel pathogenic variants that explain the underlying genetic basis for phenotypic expansion and provide strong support to apply the WES pipeline in genetic diagnosis, especially for highly heterogeneous rare diseases like skeletal dysplasia.

## Materials and Methods

### Subjects and DNA Isolation

**FIGURE 1:**
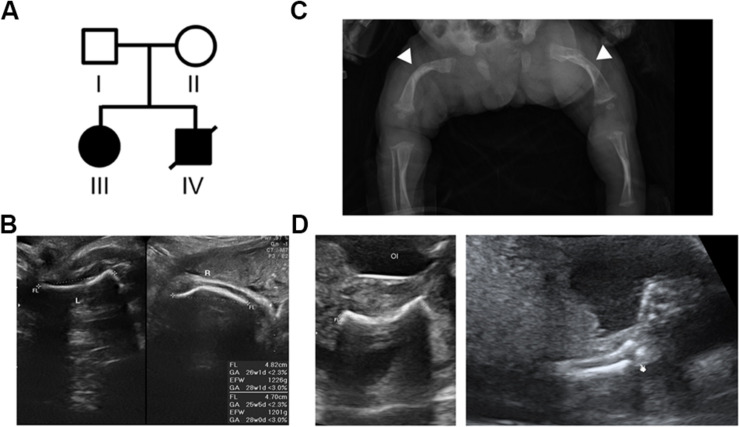
Pedigree of the family with skeletal dysplasia and their phenotypes. **(A)** Pedigree of the family with skeletal dysplasia. **(B)** Prenatal ultrasound images of patient III show both short left (L) and right (R) femurs. **(C)** Radiography of patient III after birth shows osteopenia and curvature and fractures of the femurs (arrow heads) with widening of the distal diaphysis. **(D)** Ultrasound images of the proband (patient IV) show the short and fractured femur (left) and fractured radius (right).

This study included a family with two individuals affected with skeletal dysplasia. The parents (Figure 1A, Individuals I and II) and proband (Figure 1A, Individual IV) requested a molecular genetic testing procedure for future prenatal diagnosis. The family underwent clinical examinations at the Fetal Medicine Center (FMC, Taiwan) and the Department of Pediatrics, Chi Mei Medical Center (Taiwan). Informed consent was obtained from all participants. The proband’s sibling (Figure 1A, Individual III) had a clinical diagnosis of OI after birth based on radiographic findings. Due to frequent long bone fractures, she has been on pamidronate therapy since the age of 1 year and 4 months. The parents were asymptomatic and had no family history of skeletal dysplasia. Written and signed informed consent was obtained from all subjects of this study or their legal guardians and approved by the Institutional Review Board (IRB) of the National Cheng Kung University Hospital. Written informed consent for the publication of any potentially identifiable images or data included in this article was obtained from the individuals or their legal guardians.

Genomic DNA was isolated from peripheral blood (Individuals I, II, and III) and umbilical tissue (proband, Individual IV) using QIAamp DNA Blood Mini Kit (QIAGEN, Hilden, Germany; cat. no.: #51104) supplemented with Buffer ATL (QIAGEN; cat. no.: #19076) according to the manufacturer’s instructions.

### Library Preparation, Exome Enrichment, and Sequencing

Approximately 100 ng DNA was sheared using the Covaris S2 system (Covaris Inc., Woburn, MA, United States) to generate fragmented DNA mainly between 150 and 300 bp. The fragmented DNA was used for library construction using the KAPA HyperPrep Kit (KAPA Biosystems, Wilmington, MA, United States; cat. no.: #07962312001). The quantity of library was determined using the Qubit dsDNA HS assay kit with the Qubit 2.0 Fluorometer (Thermo Scientific, Waltham, MA, United States). A total of 1,000 ng mixed DNA libraries was captured using the SeqCap EZ MedExome Enrichment Kit (Roche NimbleGen Inc., Madison, WI, United States; cat. no.: #07676581001), which targets ∼19,390 genes covering 47 Mb genomic sequences. The captured exomes were subsequently quantified by the KAPA Library Quantification Kit (KAPA Biosystems; cat. no.: #07960336001), followed by sequencing using NextSeq 550 (Illumina, San Diego, CA, United States) with NextSeq 500/550 High Output v2 kit (300 cycles: 2 × 150 bp paired-end reads) according to the protocol provided by the vendor.

### Bioinformatics Pipeline for Mapping and Variant Calling

An in-house bioinformatic pipeline was built to process sequencing reads. Briefly, the sequencing reads were first trimmed using the Trimmomatic tool (base quality ≥ Q20), followed by a quality check using the FastQC tool. Next, the trimmed reads were mapped against the human reference genome (hg19,^1^) using the BWA-MEM algorithm (Li and Durbin, 2009). The output SAM file was then converted to a BAM file, and only the unique mapped reads were used for variant calling using the Genome Analysis Toolkit (GATK) platform^2^ according to the “Best Practices” guidelines (version 4) from the Broad Institute. GATK-Mutect2 and FilterMutectCalls were conducted to identify SNVs and insertions and deletions (indels) using default parameters. The output Variant Call Format (VCF) file was annotated with various public databases, including dbSNP^3^, SIFT^4^, PolyPhen-2^5^, MutationTaster^6^, PROVEAN^7^, FATHMM^8^, gnomAD database^9^, 1000 Genomes projects^10^, and ClinVar^11^, using the ANNOVAR program (Wang et al., 2010). In addition, the minor allele frequency (MAF) was calculated from 996 Taiwanese whole-genome sequencing (WGS) VCF data (Lin et al., 2018) and used as a plugin in our pipeline to delimitate pathogenic variant(s).

### Detection of Copy Number Variation (CNV)

To analyze the regional DNA copy changes from each WES sample, the CNVkit (Talevich et al., 2016) that uses both the targeted reads and the non-specifically captured off-target reads to infer copy number evenly across the genome was applied, and this was generated by the BWA-MEM algorithm. Briefly, the target and off-target coverages of each sample were calculated based on the read counts per bin using the BAM file as input. The averages of target and off-target coverages from normal populations served as a reference. Normalization of samples to reference was performed and converted to log2 values. The segmentation was then conducted using the Circular Binary Segmentation algorithm by the default setting. Finally, a scatter plot was drawn using the normalized log2 value of each bin together with the segments.

In addition, the raw WES read counts of each exon within chr3p22.3 (31573992…36986548) were extracted and normalized to the total mapped read counts to obtain the CNV ratio within the target region. This was followed by summing up all normalized read counts in a given gene and the maternal-to-paternal gene ratio or proband-to-paternal gene ratio across the chr3p22.3, which was then plotted into a graph according to the genomic coordinates. The deleted region identified from the WES data was confirmed by aCGH using the CytoOneArray^®^ (Phalanx Biotech, Taiwan), which is designed to identify chromosomal microduplications and microdeletions, especially in the disease-related genomic regions, using the genomic DNAs from both parents and proband.

### Sanger Sequencing

The primers used for amplifying genomic regions of *CRTAP* and *MESP2* are as follows: *CRTAP*, forward sequence 5'-CAA ATG GAG TGG AAG CCG AGG T-3', reverse sequence 5'-GGG CTG TTG GGA AAA GGA CAA-3'; and *MESP2*, forward sequence 5'-GAG CCC AAG CCG CAC-3', reverse sequence 5'-GGG TTC CTT CCA TTC TCC C-3'. The PCR products were sequenced on the ABI PRISM 3130XL Genetic Analyzer.

### RNA Isolation and Quantitative RT–PCR

Total RNA was isolated from the paternal, maternal, and normal control’s buffy coat using TRIzol (Invitrogen, Carlsbad, CA, United States) and purified by ethanol precipitation. Reverse–transcription and quantitative PCR assays were performed using High Capacity cDNA Reverse Transcription kit (Applied Biosystems, Foster City, CA, United States; cat. no.: #4368813).

For quantification of *CRTAP* mRNA in pre–mRNA and mature mRNA levels, KAPA SYBR Fast qPCR kit (KAPA Biosystems, Wilmington, MA, United States; cat. no.: #KK4973) was used, and the beta actin (*ACTB*) level was used as an internal control. All reactions were run on the SYBR program at the default setting using an Applied Biosystems StepOnePlus Real–Time PCR system in a 96–well plate format. Primer sequences for specific genes are as follows: for pre–mRNA of *CRTAP*, forward sequence 5'-GAC CAC ACT CCA GAA GGA GC-3', reverse sequence 5'-ACT TCA GGG AGG ACT CAG CC-3'; for mature mRNA of *CRTAP*, forward sequence: 5'-GAC AAG GTC ATG CAG CAG AA-3', reverse sequence 5'-CTG AAC GCC AAG AGG AAG TC-3'; and for *ACTB*, forward sequence 5'-GCC CTG AGG CAC TCT TCC A-3', reverse sequence 5'-CGG ATG TCC ACG TCA CAC TT-3'.

## Results

### Case Presentation

This study included a family with two affected offspring with skeletal disorders, while both the parents are aged in their 20s, healthy (Figure 1A, Individuals I and II), and without a family history of skeletal disorders. The proband’s elder sister (Figure 1A, Individual III) presented short limbs on prenatal ultrasound images (Figure 1B) and was diagnosed with OI after birth because of curvature and fractures of bilateral femurs on X-ray (Figure 1C). Due to frequent long bone fractures, she received regular intravenous pamidronate therapy from the age of 1 year and 4 months, and the follow-up bone mineral density and fracture rates significantly improved. The aborted proband (Figure 1A, Individual IV) exhibited short long bones and multiple fractures on ultrasound images at 21 weeks of gestational age (Figure 1D), indicating symptoms which are related to OI and more severe than that observed in his sibling. The pedigree of normal parents with two affected offspring implies an autosomal recessive inheritance underlying the skeletal dysplasia within this family.

### Identification of Candidate Mutations in Proband Using WES

To identify the genetic cause of skeletal disorder in this family, we first screened *COL1A1* and *COL1A2* genes using Sanger sequencing, the two most common genes that are known to cause OI, but this revealed no mutations. Next, we applied WES for the trio study using the proband’s umbilical DNA and parents’ blood DNA to examine the genetic basis of the skeletal disorder. The sequencing was conducted on the illumine NextSeq 550 platform at a sequencing depth of ∼100×. The sequencing reads covered 99.8% of human exonic regions, while the uniformity and on-target rate were 97.0 and 82.8%, respectively. The mapping reads were subjected to the GATK (McKenna et al., 2010) for variant calling with the default parameters of the Mutect2 tool.

**FIGURE 2:**
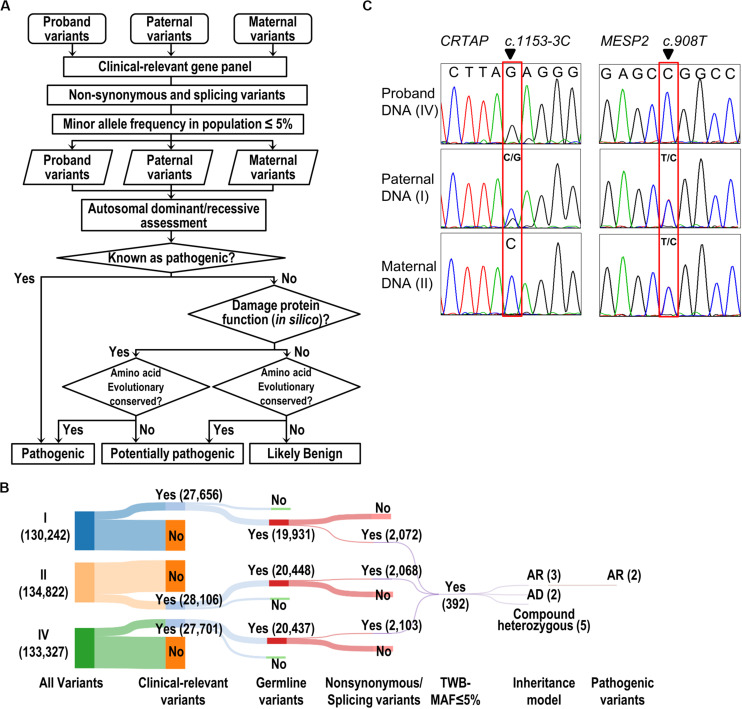
Identification and validation of pathogenic variants. **(A)** The flow of the pathogenic variant discovery pipeline. The genetic variants were obtained by GATK variant calling algorithm. The SIFT, PolyPhen-2, and MutationTaster were used for prediction of the effect of a variant on the damage of protein function. Human Splicing Finder and dbscSNV were used to predict the consequence of splicing variant. The evolutionary conservation was scored by the phastCons tool. **(B)** The Sankey diagram shows the reduced volume of variants from the trio study (Individuals I, II, and IV). The number of variants is shown in parentheses. **(C)** Validation of WES results by Sanger-seq at *CRTAP* and *MESP2* loci.

With the attempt to identify causative genetic mutation(s), we developed a “pathogenic variant discovery pipeline” (Figure 2A) to annotate the sequence variants obtained from exome sequencing. The number of variants generated after each filtering step in our pipeline (Figure 2A) is depicted and shown in the Sankey diagram (Figure 2B). In general, the WES procedure discovers 130–134 k variants per individual. Among all the variants, we focused on those belonging to the clinically relevant genes derived from the OMIM database and obtained around 27–28 k variants per individual (Figure 2B). Next, we selected variants that lead to amino acid changes or splicing errors and reduced the number to ∼2 k variants per individual. This was followed by filtering the remaining variants using a MAF less than 5% in the Taiwanese population (data derived from Taiwan Biobank, Academia Sinica, Taiwan (Lin et al., 2018), from which we obtained 392 variants merged from all three family members. We further filtered variants to meet with the recessive inheritance according to the family transmission pattern. Finally, we evaluated the predicted impacts of variants on the gene product or protein function, together with the conservation of genomic positions of each variant, and therefore pinpointed two candidate variants: a splicing variant (c.1153-3C > G) in the *CRTAP* gene and a missense variant (c.908T > C, p.Leu303Pro) in the *MESP2* gene (Figure 2B and Table 1).

**TABLE 1:** Detailed information of variants in *CRTAP* and *MESP2* genes.

Gene	Coordinate^a^	Mutation	Type	Amino acid change	Population MAF (Taiwanese)^b^	Population MAF (EAS)^c^
*CRTAP*	chr3:33183884	NM_006371.4 c.1153-3C > G	Splicing altering	-	0.001	<0.0001
*MESP2*	chr15:90320496	NM_001039958.2 c.908T > C	Missense	p.Leu303Pro	0.034	0.020

*^*a*^Genomic coordinate from GRCh37 (also known as hg19). ^*b*^The population MAF was derived from Taiwan Biobank (Academia Sinica, Taiwan). MAF, minor allele frequency. ^*c*^The population MAF was derived from the 1000 Genomes Project. EAS, East Asian.*

The Sanger sequencing was performed to confirm that the proband was homozygous at the position c.1153-3C > G in *CRTAP* gene and c.908T > C in *MESP2* gene (Figure 2C). The father carries heterozygous variants in *CRTAP* (c.1153-3C > G) and *MESP2* (c.908T > C), while the mother harbors wild-type *CRTAP* and a heterozygous variant in *MESP2* (c.908T > C) (Figure 2C).

### Confirmation of Causing Compound Heterozygous Mutations of CRTAP (c.1153-3C > G/deletion) in the Proband

**FIGURE 3:**
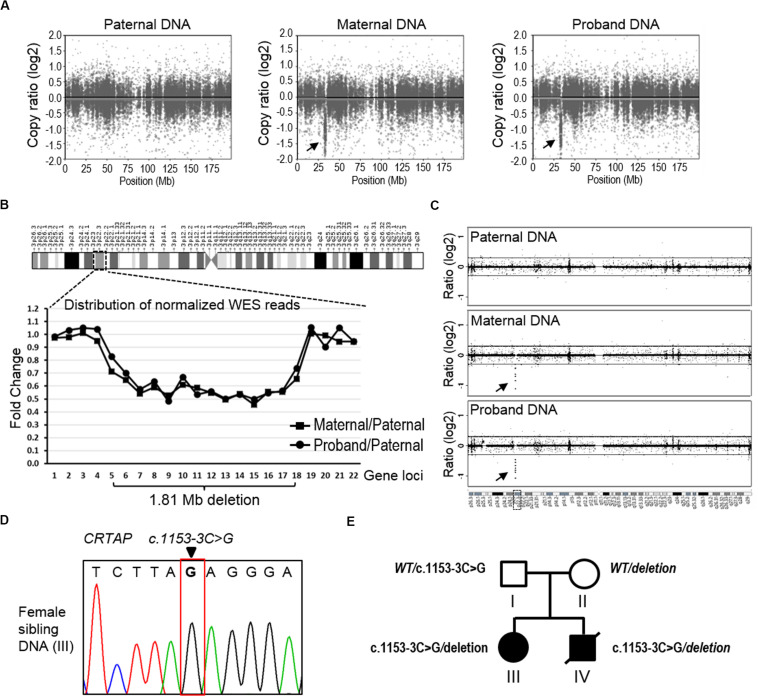
The detection of copy number variation of the *CRTAP* gene. **(A)** The CNVkit was conducted to call CNV using WES data from the father (left), mother (middle), and proband (right). The scatter plot of chromosome three shows a ∼1.81 Mb deletion at the p22.3 region in both maternal and proband samples. The arrows indicate discrete copy number segments across chromosome 3. **(B)** The distribution of normalized WES reads shows the 1.81 Mb deletion that covers 14 gene loci across the chr3p22.3 in the maternal and proband DNA. The ratio of normalized read counts, maternal/paternal or proband/paternal, of each gene was plotted in the graph according to the genomic loci of the successive 22 genes in chr3p22.3. *CRTAP* is the no. 13 gene (*X*-axis). **(C)** The data of aCGH show a deletion in the maternal and proband DNA in the chr3p22.3 region. The arrows indicate the region of detected deletion. **(D)** Sanger-seq at the *CRTAP* locus from the elder sibling of the proband (Individual III). **(E)** The summary of mutation and inherited genotype of *CRTAP* (c.1153-3C) in the family with osteogenesis imperfecta.

Notably, we found that the mother showed the homozygous wild type at position c.1153-3C of the *CRTAP* gene. As the proband showed homozygous paternal mutant alleles, it is hard to explain the missing allele transmitted from mother to the offspring (Figure 2C). We suspect that the mother may have a deletion within this region. To analyze CNV, we applied the CNVkit, a tool developed specifically to call CNV using data from WES or targeted-seq (Talevich et al., 2016), to search for the explanation for the missing maternal allele. Results from the CNVkit clearly showed that the mother carried a ∼1.81 Mb deletion at chr3p22.3 (hg19, chr3:g.32398837_34210906del), and she passed this deletion to the proband, while the father did not have any CNV at this region (Figure 3A). In parallel, we plotted the copy number ratios using normalized read counts from the mother or proband compared to that of the father’s (Figure 3B). Consistent with the results from the CNVkit, we found both mother and proband exhibited this 1.81 Mb deletion covering 14 gene loci on chr3p22.3 (Figure 3B). The regional deletion was confirmed by aCGH (Figure 3C). Collectively, these data showed that the father is a carrier of the *CRTAP* splicing mutation (c.1153-3C > G), while the mother carries a 1.81 Mb deletion on the chr3p22.3 region that contains the *CRTAP* gene. The proband thus has inherited compound heterozygote mutations that are one deletion and one SNV of the *CRTAP* gene from the parents. Notably, the identified SNV and large deletion of *CRTAP* have not hitherto been reported in the literature, nor have they been collected in the Human Gene Mutation Database^12^ (Supplementary Table 1), thus indicating that the compound heterozygous variants (c.1153-3C > G/deletion) represent novel *CRTAP* mutations.

In addition, we examined the elder sibling of the proband who was diagnosed with OI and found that she harbored the same *CRTAP* compound heterozygous variants (c.1153-3C > G/deletion, Figure 3D), indicating the disease-causing role of the *CRTAP* mutations (Figure 3E).

### Exclude the Involvement of MESP2 Mutation (c.908T > C) in the Clinical Phenotype of the Proband

The missense mutation of *MESP2* (c.908T > C, p.Leu303Pro) is located in a critical functional domain, and the affected amino acid of MESP2 (p.Leu303) is highly conserved across species (data not shown), and this was also indicated by the phastCons analysis (0.841) (Siepel et al., 2005) on the conservation of genomic position (Table 2). However, the mutation was predicted to be tolerated by PolyPhen-2_HDIV (0.331) and PROVEAN (−1.83), has been observed in 23 healthy adults in homozygous state, and reported a MAF of 0.0047 in the gnomAD exomes database. Furthermore, *MESP2* (c.908T > C, p.Leu303Pro) was classified as a benign variant based on the ClinVar database or according to the American College of Medical Genetics and Genomics (ACMG) guidelines (Table 2). Taken together, we thus excluded the involvement of the *MESP2* (c.908T > C) mutation in the clinical phenotype of the proband and dropped it from further analysis.

**TABLE 2:** Prediction of functional consequence caused by *CRTAP* and *MESP2* mutations and the pathogenicity classification^a^.

Gene Mutation		*CRTAP NM_006371.4 c.1153-3C > G*	*MESP2 NM_001039958.2 c.908T > C*
	
Category	Tool	Score (consequence)	Score (consequence)
Splicing altering effect	dbscSNV_ADA	0.999 (splicing altering)	–
	dbscSNV_RF	0.932 (splicing altering)	–
	Human splicing finder	Broken WT acceptor site	–
Protein function	SIFT	–	0.02 (deleterious)
	PolyPhen-2	–	0.33 (benign)
	PROVEAN	–	−1.83 (neutral)
	FATHMM	–	−2.17 (deleterious)
Gene/protein function	MutationTaster	Disease causing	Disease causing
Nucleotide conservation	PhastCons	0.911 (conserved)	0.841 (conserved)
Variant classification	ACMG	Pathogenic PS3, PM2, PM3, PM4	Benign PM1, BS1, BS2, BP6

*^*a*^Variant classification is based on the criteria addressed in ACMG standards and guidelines. dbscSNV, database of splicing consensus single-nucleotide variants; SIFT, the sorting intolerant from tolerant; PolyPhen-2, polymorphism phenotyping v2; PROVEAN, protein variation effect analyzer; FATHMM, functional analysis through hidden markov models; –, not applicable.*

### Biological Effect and Clinical Significance of CRTAP Mutation

**FIGURE 4:**
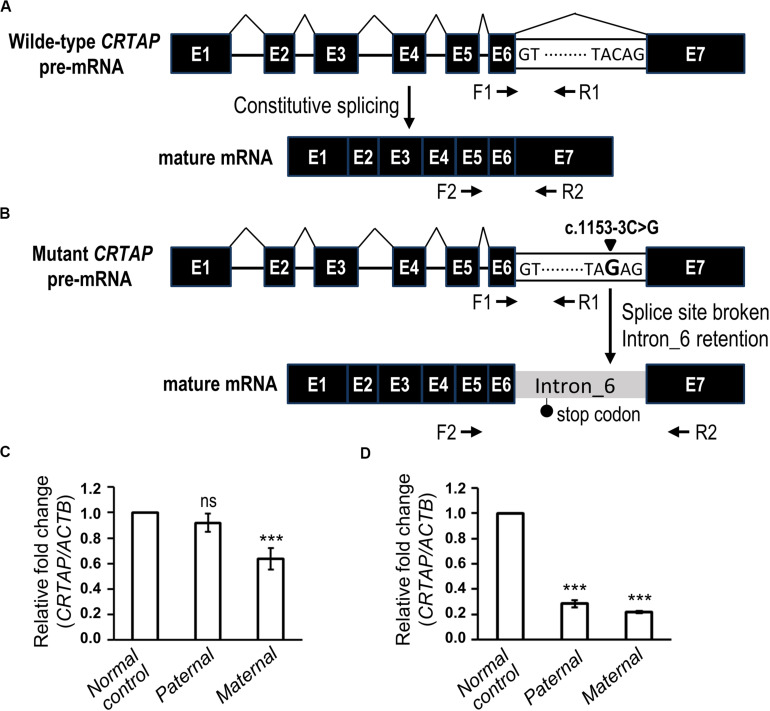
The biological effect of the splice variant of *CRTAP* (c.1153-3C > G). **(A,B)** Scheme depicting the consequence of RNA splicing of wild-type *CRTAP* pre-mRNA **(A)** or mutant *CRTAP* (c.1153-3C > G) pre-mRNA **(B)**. The mutation of the consensus splice acceptor site of *CRTAP* results in a broken splice site and intron six retention and generates a premature termination codon (stop codon). The arrows indicate the primers designed for amplification of pre-mRNA (F1/R1) or mature mRNA of *CRTAP* (F2/R2). **(C,D)** Quantitative PCR analysis shows the relative expression of the *CRTAP* pre-mRNA level **(C)** and mature mRNA level **(D)** of the paternal, maternal, or normal control sample. Error bars represent SEM, *N* = 3. *P*-values are calculated with a two-tailed *t*-test. ***P < 0.001; ns, not significant.

The genomic position of the *CRTAP* mutation (c.1153-3C) was highly conserved as shown by phastCons (0.911) analysis (Siepel et al., 2005), and the mutation was predicted to be “disease causing” using the MutationTaster tool (Table 2). To determine the effect of the splicing variant of the *CRTAP* gene, the Human Splicing Finder and Database of Splicing Consensus Single-Nucleotide Variants (dbscSNV) were used to predict the consequence of *CRTAP* (c.1153-3C > G) mutation. The *CRTAP* c.1153-3C is located at the -3 position upstream from exon seven in intron six (Figure 4A). The C > G at the -3 position was predicted to damage the splicing process and break the original splicing acceptor site by dbscSNV and Human Splicing Finder, respectively, which resulted in intron six retention (Table 2 and Figure 4B). Sequence analysis indicates that the intron retention leads to a premature termination codon after five additional amino acids downstream from the junction of exon–intron six, and thus, it may induce nonsense-mediated mRNA decay (NMD) (Isken and Maquat, 2007; Hug et al., 2016). On the other hand, the deletion of one allele of the *CRTAP* gene has been shown to decrease the expression level of mature mRNA (Morello et al., 2006).

It is expected that deletion of *CRTAP* results in reduction of both pre-mRNA and mature mRNA, whereas the intron retention-mediated NMD is supposed to decrease mature mRNA without changing the pre-mRNA level. To this end, we examined the expression levels of pre-mRNA (Figures 4A,B, F1 and R1) and mature mRNA (Figures 4A,B, F2 and R2) of *CRTAP* using quantitative RT-PCR in the paternal sample harboring the heterozygous *CRTAP* c.1153-3C > G mutation and the maternal sample harboring the heterozygous *CRTAP* deletion. In line with our prediction, the paternal sample expressed the same level of *CRTAP* pre-mRNA as the normal control (Figure 4C), while the paternal mature mRNA level was dramatically reduced to the same level as the maternal sample (Figure 4D). In contrast, both *CRTAP* pre-mRNA and mRNA levels of the maternal sample were lower compared to those of the normal control (Figures 4C,D). These data demonstrated that the splicing mutation in the *CRTAP* gene (c.1153-3C > G) results in a splicing error and a dramatic decrease of *CRTAP* mRNA.

According to the guidelines of sequence variant interpretation made by the ACMG (Richards et al., 2015), we confirmed that the splicing mutation in the *CRTAP* gene (c.1153-3C > G) significantly decreased the gene product [as a strong evidence of pathogenicity 3 (PS3)], resulted in length change of the gene product [as a moderate evidence of pathogenicity 4 (PM4)], and were detected in trans with a pathogenic variant at very low frequency (as evidence of PM2 and PM3). Therefore, the *CRTAP* c.1153-3C > G mutation was classified as a pathogenic variant (Table 2).

## Discussion

Due to the strong clinical and genetic heterogeneities, searching for the causative mutations of skeletal dysplasia remains a task with big challenges. With the advance of next-generation sequencing (NGS) technology, attempts have been made to implement and apply it to offer a comprehensive strategy in clinical practice (Liu et al., 2019; Yang et al., 2019). In the current study, through integration of WES and molecular analyses, we identified novel pathogenic compound mutations in patients affected with OI: a splicing error-causing mutation (NM_006371.4:c.1153-3C > G) and a large deletion in the *CRTAP* gene (hg19, chr3:g.32398837_34210906del). The pathogenicity of the novel mutation was also evaluated according to the latest guidelines of sequence variant interpretation made by ACMG (Table 2).

OI is a collective term for a group of rare connective tissue syndromes characterized by extremely fragile bones. It has been reported that *COL1A1* and *COL1A2* mutations account for around 90% of all cases of OI in an autosomal dominant manner (Van Dijk and Sillence, 2014), while the remaining mutations of the *CRTAP* (Type VII, OMIM #610682) and *P3H1* (Type VIII, OMIM #610915) genes are responsible for rare and severe to intermediate OI in an autosomal recessive manner (Byers and Pyott, 2012). Previous studies demonstrated that CRTAP protein is required for 3-hydroxylation of proline 986 residue of collagen α1(I) (Morello et al., 2006), and its deficiency leads to defects of collage type I biosynthesis through disrupting chain alignment initiation and helical folding (Pyott et al., 2011). Among the reported 23 *CRTAP* pathogenic mutations, the majority of them are SNVs (14) and small indels (8) and result in premature termination codons, while the remaining one is a gross deletion of the promoter to the intron 1 region in the *CRTAP* gene (Supplementary Table 1). The data suggest that the amount of gene products (protein) is the key factor influencing the collage type I biosynthesis. Here, we report the findings of novel compound heterozygous *CRTAP* mutations (c.1153-3C > G/deletion) identified in the proband and his sibling. By using quantitative RT-PCR, our results revealed a dramatic loss of *CRTAP* mRNA expression through both chromosomal deletion of one allele and probably the NMD of mRNA from the splicing variant. It has been shown that null mutations with a dramatic reduction of *CRTAP* gene products as well as 3-hydroxylation of the Pro986 residue of collagen α1(I) cause severe OI (Marini et al., 2010). Given that the CRTAP protein mutually stabilizes P3H1 in the collagen prolyl 3-hydroxylation complex (Chang et al., 2010), the reduction of CRTAP expression thus has great impact on the collage type I biosynthesis and leads to the observed OI phenotype. Consistent with clinical indications, the sibling was diagnosed as OI with mitral regurgitation and developmental delay, who is now under pamidronate infusion therapy. Notably, the mother harbored a heterozygous 1.81 Mb deletion in chr3p22.3 that covers 14 gene loci, but she is clinically asymptomatic. Among genes within this region, only two genes, *CRTAP* and *GLB1*, have been reported to be associated with genetic diseases, both of which are manifested in an autosomal recessive manner. As the mother is a heterozygote for the deletion in chr3p22.3, our WES data confirmed that she does not carry any pathogenic variant in the other allele of the *GLB1* gene, explaining the observation of a clinically normal phenotype.

The majority of variants in the human population are rare due to *de novo* mutations occurring in each generation (1000 Genomes Project Consortium et al., 2012, 2015). Most of these rare variants are non-functional or neutral, and as such, they could accumulate in human populations (Harpak et al., 2016; Lappalainen et al., 2019). In line with the previous findings, we analyzed the variants in the Taiwanese population (Lin et al., 2018) and found similar features. Among 38.9 million variants discovered from WGS in 996 Taiwanese individuals, 31.9 million variants (81.8%) are of low to rare frequency (MAF < 5%). Nevertheless, there are 3,008 (0.0094%) known disease-causing variants that correspond to 2,354 genes in these low- to rare-frequency variants. Although purifying selection may remove a number of deleterious variants from populations (Karczewski et al., 2019), some deleterious variants will be retained and predispose to a disease in an autosomal recessive manner. Taking skeletal dysplasia as an example, we found that 64 out of 3,008 known disease-causing variants are involved in skeletal dysplasia, while 54 variants (84.4%) cause skeletal dysplasia by autosomal recessive inheritance. Most importantly, the MAF of the *CRTAP* mutation identified in this study is 0.1% in Taiwanese people; therefore, we shall take extra precautions to evaluate low-MAF allele (0.1-5%) for the possible pathogenicity leading to autosome recessive diseases in the population. The expanded carrier screening is of importance to identify such variants in couples before pregnancy. Once the couples are found to be at risk for transmitting certain types of diseases, *in vitro* fertilization and preimplantation genetic testing (PGT) for monogenic disorders may be considered to stop transmitting deleterious variants into the next generation (Daar et al., 2018; Fu et al., 2019; van der Schoot et al., 2019).

In summary, our data uncover novel compound pathogenic variants that explain the underlying genetic basis for OI in a Taiwanese family. We not only expand the mutation scale of skeletal dysplasia but also provide a molecular insight into the effects of these mutations. In addition, we hope to draw more attention to the abundant low-frequency variants leading to autosomal recessive diseases. The proper application of WES technology in the expanded carrier test shall provide indispensable value to reduce the detrimental effect of these variants on the human population.

## Data Availability Statement

The raw data supporting the conclusions of this article will be made available by the authors, without undue reservation, to any qualified researcher.

## Ethics Statement

The studies involving human participants were reviewed and approved by IRB of National Cheng Kung University Hospital. Written informed consent to participate in this study was provided by the participants’ legal guardian/next of kin.

## Author Contributions

Y-AT and HS conceived and designed this study. Y-AT, C-MC, and HS processed, analyzed, and interpreted the data. L-YW, I-WL, and W-HT contributed in the assessment and interpretation of the clinical symptoms and data. I-WL and W-HT contributed in the recruitment of patients in the hospital. Y-AT and HS wrote the manuscript. All authors contributed to the improvement of the manuscript and read the final version of the manuscript.

## Conflict of Interest

The authors declare that the research was conducted in the absence of any commercial or financial relationships that could be construed as a potential conflict of interest.

## Footnotes

1 http://genome.ucsc.edu

2 https://software.broadinstitute.org/gatk/

3 https://www.ncbi.nlm.nih.gov/projects/SNP/

4 https://sift.bii.a-star.edu.sg/

5 http://genetics.bwh.harvard.edu/pph2/

6 http://www.mutationtaster.org/

7 http://provean.jcvi.org/index.php

8 http://fathmm.biocompute.org.uk/

9 https://gnomad.broadinstitute.org/

10 https://www.internationalgenome.org/

11 https://www.ncbi.nlm.nih.gov/clinvar/

12 http://www.hgmd.cf.ac.uk/ac/index.php
