# Supplementary material for: Novel Compound Heterozygous Mutations in CRTAP Cause Rare Autosomal Recessive Osteogenesis Imperfecta
Source: Front Genet. 2020 Aug 14;11:897. doi: 10.3389/fgene.2020.00897 (PMC7457090; doi:10.3389/fgene.2020.00897)
Supplement: Supplementary file 1 [file Table_1.DOCX]

Supplementary Table 1. Detailed information of pathogenic and likely pathogenic variants of *CRTAP* gene^a^.

| **No** | **Coordinate**^b^ | **Mutation** | **Type** | **Variant class** | **dbSNP** | **HGMD**^c^ **accession #** |
| --- | --- | --- | --- | --- | --- | --- |
| 1 | Chr3:33155572 | c.3G>A  (p.Met1Ile) | Missense  (Start-loss) | Disease-causing | rs72659357 | CM067661 |
| 2 | Chr3:33155607 | c.38C>A (p.Ala13Glu) | Missense | Disease-causing | rs137853938 | CM098071 |
| 3 | Chr3:33155687 | c.118G>T (p.Glu40Ter) | Nonsense | Disease-causing | rs863225043 | CM151919 |
| 4 | Chr3:33155702 | c.133G>T (p.Glu45Ter) | Nonsense | Disease-causing | -- | CM150453 |
| 5 | Chr3:33155767 | c.198C>A (p.Tyr66Ter) | Nonsense | Disease-causing | rs137853939 | CM098070 |
| 6 | Chr3:33155769 | c.200T>C (p.Leu67Pro) | Missense | Disease-causing | rs72659358 | CM086807 |
| 7 | Chr3:33156038 | c.469A>G (p.Lys157Glu) | Missense | Disease-causing | rs137853942 | CM098072 |
| 8 | Chr3:33161925 | c.561T>G (p.Tyr187Ter) | Nonsense | Disease-causing | rs387907334 | CM1210078 |
| 9 | Chr3:33171463 | c.826C>T (p.Gln276Ter) | Nonsense | Disease-causing | rs72659361 | CM067660 |
| 10 | Chr3: 33156041 | c.471+1G>C | Splicing altering | Disease-causing | rs72659359 | CS067817 |
| 11 | Chr3: 33156042 | c.471+2C>A | Splicing altering | Disease-causing | rs137853943 | CS090289 |
| 12 | Chr3:33160815 | c.472-1021C>G | Splicing altering | Disease-causing | rs72659360 | CS067818 |
| 13 | Chr3:33161922 | c.624-64A>G | Splicing altering | Likely disease-causing | rs35357409 | CS1513843 |
| 14 | Chr3:33174045 | c.923-2A>G | Splicing altering | Disease-causing | rs137853947 | CS098073 |
| 15 | Chr3:33155593-33155600 | c.24_31delCGCGGCGC (p.Ala10Serfs) | Small deletion | Disease-causing | rs755750808 | CD086126 |
| 16 | Chr3:33155973-33155973 | c.404delG  (p.Ser135Thrfs) | Small deletion | Disease-causing | rs137853941 | CD0910233 |
| 17 | Chr3:33171441-33171446 | c.804_809delAGAAGT (p.Glu269 _Val270del) | Small deletion | Disease-causing | rs137853945 | CD118518 |
| 18 | Chr3: 33171514 | c.879del (p.Phe293Leufs) | Small deletion | Disease-causing | rs72659362 | CD068294 |
| 19 | Chr3:33155591-33155592 | c.21_22dupGG  (p.Ala8Glyfs) | Small insertion | Disease-causing | rs137853936 | CI0910232 |
| 20 | Chr3:33155862-33155863 | c.278_293dupAGCCCGAGCCCGCCGC  (p.Gly99Alafs) | Small insertion | Disease-causing | rs137853940 | CI067898 |
| 21 | Chr3:33155687 - 33155702 | c.118_133delGAGCTGATGCCGCTCGinsTACCC (p.Glu40Tyrfs) | Small indel | Disease-causing | rs387907333 | CX129271 |
| 22 | Chr3:33171459-33171463 | c.822_826delAATAC  insT | Small indel | Disease-causing | rs137853946 | CX086128 |
| 23 | Chr3:33153893-33157632 | c.-1677_471+1592del | Gross deletion | Disease-causing | -- | CG134714 |
| 24 | Chr3:33183884 | c.1153-3C>G | Splicing altering | Disease-causing^d^ | rs201554363 | -- |
| 25 | Chr3:32398837-34210906 | g.32398837_34210906del | Gross deletion | Disease-causing^d^ | -- | -- |

^a^ --, not applicable. indel, insertion and deletion.

^b^ Genomic coordinate from GRCh37 (also known as hg19).

^c^ HGMD, The Human Gene Mutation Database.

^d^ Reported in this study.
